# Psychiatric Manifestations in Children and Adolescents with Inherited Metabolic Diseases

**DOI:** 10.3390/jcm13082190

**Published:** 2024-04-10

**Authors:** Valentina Baglioni, Fabiola Bozza, Giuliana Lentini, Annachiara Beatrice, Noemi Cameli, Elisa Maria Colacino Cinnante, Arianna Terrinoni, Francesca Nardecchia, Francesco Pisani

**Affiliations:** Child Neurology and Psychiatry Unit, Department of Human Neuroscience, Sapienza University, Via dei Sabelli 108, 00185 Rome, Italy; valentina.baglioni@uniroma1.it (V.B.); giuliana.lentini@uniroma1.it (G.L.); annachiara.beatrice@uniroma1.it (A.B.); noemi.cameli@uniroma1.it (N.C.); elisamaria.colacinocinnante@uniroma1.it (E.M.C.C.); ariannaterrinoni00@gmail.com (A.T.); francesca.nardecchia@uniroma1.it (F.N.); francesco.pisani@uniroma1.it (F.P.)

**Keywords:** neuropsychiatric manifestations, neuropsychiatric symptoms, metabolic diseases, inherited metabolic disorders, inborn errors of metabolism, IEMs

## Abstract

**Background**: Inherited metabolic disorders (IEMs) can be represented in children and adolescents by psychiatric disorders. The early diagnosis of IEMs is crucial for clinical outcome and treatment. The aim of this review is to analyze the most recurrent and specific psychiatric features related to IEMs in pediatrics, based on the onset type and psychiatric phenotypes. **Methods:** Following the PRISMA Statement, a systematic literature review was performed using a predefined algorithm to find suitable publications in scientific databases of interest. After removing duplicates and screening titles and abstracts, suitable papers were analyzed and screened for inclusion and exclusion criteria. Finally, the data of interest were retrieved from the remaining articles. **Results:** The results of this study are reported by type of symptoms onset (acute and chronic) and by possible psychiatric features related to IEMs. Psychiatric phenomenology has been grouped into five main clinical manifestations: mood and anxiety disorders; schizophrenia-spectrum disorders; catatonia; eating disorders; and self-injurious behaviors. **Conclusions:** The inclusion of a variety of psychiatric manifestations in children and adolescents with different IEMs is a key strength of this study, which allowed us to explore the facets of seemingly different disorders in depth, avoiding possible misdiagnoses, with the related delay of early and appropriate treatments.

## 1. Introduction

Psychiatric disorders in children and adolescents have often been reported as features of inherited metabolic disorders (IEMs).

Many children affected by metabolic disorders, indeed, could present early-onset concomitant psychiatric symptoms [1,2].

Metabolic disorders are conditions in which an altered metabolic pathway leads to the accumulation or defect of particular compounds (such as enzymes or proteins). Breakdown of these metabolic processes can interfere with Central Nervous System (CNS) development and typical functioning and, depending on its severity, lead to gross neurodevelopmental disruption, seizures and coma (severe disruption), or cognitive impairments, behavioral disorders, and psychiatric conditions such as psychosis (mild disruption) [3,4].

Both interruptions of late neurodevelopmental processes and chronic or acute disruption of excitatory/inhibitory or monoaminergic neurotransmitter systems can explain the correlation between IEMs and CNS damage [2]. A lot of psychiatric disorders can develop from an underlying metabolic condition, most often giving rise to symptoms which can also precede neurological lesions [2].

The onset of IEMs can be characterized by somatic, neurological, or cognitive symptoms but also by psychiatric symptomatology [5]. Psychiatric manifestations can be isolated before the onset of the other symptoms mentioned. Most treatments are more effective in the psychiatric phase of a disease, before the occurrence of irreversible neurological injuries [6].

Many psychiatric disorders arise during childhood and adolescence; thus, it can be difficult to distinguish between a primary psychiatric disorder and a psychiatric disorder secondary to IEMs. For this reason, it is very important for psychiatrists to be aware of the possible metabolic conditions underlying psychiatric presentations, in order to detect any potential IEM as soon as possible [7].

Inborn errors of the metabolism are a heterogeneous group of disorders that can be inherited or occur as a result of the spontaneous mutation of genes that code for proteins involved in the metabolism. Most of them are inherited as autosomal recessive. In a small percentage of cases, they can be inherited as autosomal dominant and X-linked. In addition, environmental, epigenetic, and microbiome factors play etiologic roles in IEMs [8,9,10,11].

Despite the fact that each inborn error of the metabolism is rather rare seen individually, it must be emphasized that, overall, such conditions are quite frequent as they occur in 1 in 2500 births. Inborn errors of the metabolism result in a dysfunction of the metabolic pathways involved in the degradation or storage of carbohydrates, fatty acids, and proteins [12,13,14,15].

The early diagnosis of IEMs plays an important role in the clinical outcome of the patients affected, to avoid metabolic decompensation and provide adequate genetic counseling and specific treatment when available (for some conditions, enzymatic replenishment or dietary changes might be therapeutic) [6]. Failure to recognize the underlying metabolic conditions in a timely manner can result in irreversible CNS damage due to the delay in introducing the appropriate treatment [4].

## 2. Aim

In this systematic review, we provide an up-to-date overview of the literature with a comprehensive analysis of different psychiatric manifestations in pediatric patients with IEMs. The results of this study are reported according to two criteria, by type of symptoms’ onset during pediatric age and by possible psychiatric features related to IEMs, with the aim to be supportive during clinical practice in order to distinguish important red flags in psychiatry for IEMs and suggest timely analyses and investigations during differential diagnosis among psychiatric and neurological features in children and adolescents.

Psychiatric phenomenology has been grouped according to five main clinical manifestations: mood and anxiety disorders; schizophrenia-spectrum disorders; catatonia; eating disorders; and self-injurious behaviors (SIBs).

Moreover, further clinical information has been reported concerning the time of onset of these challenging symptoms.

## 3. Materials and Methods

**Figure 1:**
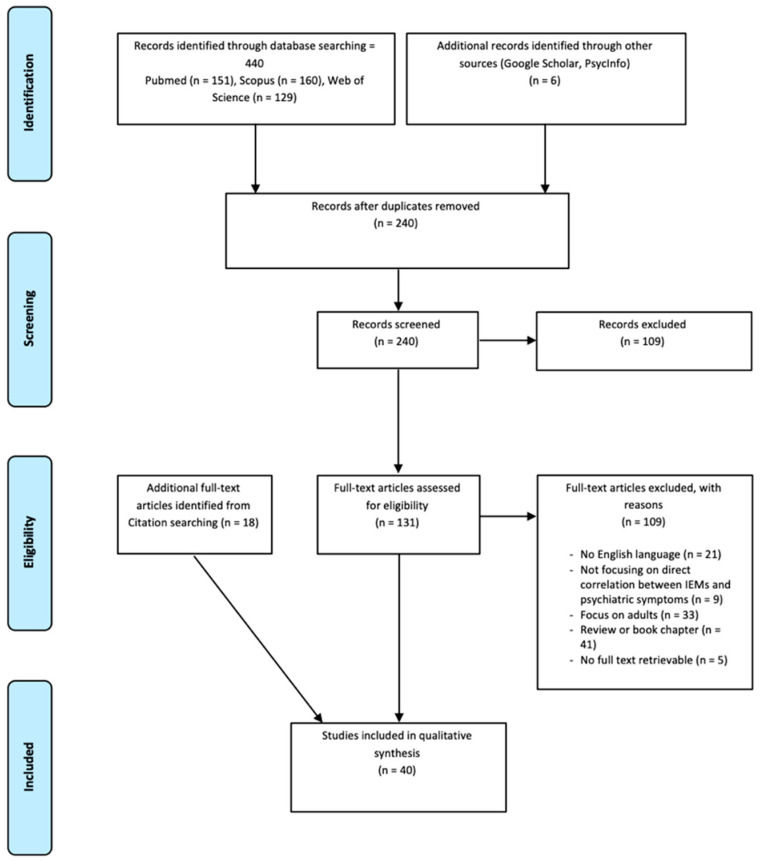
Flow-chart of the systematic review.

To investigate the direct correlation between IEMs and psychiatric disorders, a systematic literature review was performed according to the Preferred Reporting Items for Systematic Reviews and Meta-Analysis (PRISMA) Statement [16]. The review protocol was registered in the PROSPERO database (CRD42024526354). Figure 1 shows the PRISMA flow diagram, in which the selection process is described in detail.

Studies’ retrieval and the selection process of eligible articles to be included in the current work followed three steps. Firstly, a predefined algorithm was defined and used to search for suitable publications in scientific databases of interest. Subsequently, duplicates were removed, and titles and abstracts of the retrieved article were first screened: the papers that passed the title and abstract screening were further analyzed and screened according to predefined inclusion and exclusion criteria. Finally, data of interest were extracted from the remaining articles.

### 3.1. Database Search Strategy

We searched the PubMed (https://pubmed.ncbi.nlm.nih.gov/ accessed on 19 February 2024), Scopus (https://www.scopus.com/search/ accessed on 19 February 2024), and Web of Science (http://www.webofscience.com/ accessed on 19 February 2024) databases for publications up until February 2024, using the following algorithm: (“neuropsychiatric manifestation*” OR “psychiatric manifestation*” OR “psychiatric symptom*” OR “neuropsychiatric symptom*”) AND (“metabolic disease*” OR “inherited metabolic disease*” OR “inherited metabolic disorder*” OR “inborn errors of metabolism” OR “IEMs”). This research led us to a total of 440 initial hits.

### 3.2. Literature Search Strategy and Study Eligibility

After article retrieval through the search algorithm and the addition of articles obtained from other sources (Google Scholar and PsycInfo databases), all duplicates were removed, and all articles were considered only once. The selected publications then underwent title and abstract screening to exclude articles that were not relevant to the topic. Subsequently, the remaining articles were checked according to predefined inclusion and exclusion criteria. The inclusion criteria were the following: (a) interventional or observational studies (both retrospective and prospective studies); (b) case reports and case series; and (c) articles reporting the direct correlation between IEMs and psychiatric symptoms. The exclusion criteria were the following: (a) studies written in languages other than English; (b) reviews, letters to the editor, commentaries, meeting abstracts, book chapters, dissertations, study protocols, and seminars; and (c) animal models. When the full text was not retrievable, the article in question was excluded. The reference section of the articles that survived the application of the inclusion and exclusion criteria was checked to search for additional relevant works in the literature: whenever relevant citations were found, these publications underwent our study’s eligibility process.

The selection process was carried out by two independent reviewers: in case of disagreement, the reviewers discussed their views until a consensus was reached. When necessary, consensus was pursued by involving a third reviewer.

### 3.3. Study Inclusion

From the initial database search, a total of 440 potentially eligible articles were retrieved; to this amount, 6 articles obtained from other sources were added. Subsequently, duplicates were removed, and a total of 240 manuscripts were then screened by reading the title and abstract. One hundred and thirty-one papers survived the first step. The inclusion and exclusion criteria were then applied for further screening of the articles, and additional articles were identified from citation searching: of these, 40 fulfilled all the predefined criteria and were included in the present systematic review.

Table 1 reports the articles included in this systematic review.

## 4. Results

### 4.1. Epidemiology

The phenomenological classification of psychiatric disorders can be found both in the *Diagnostic And Statistical Manual Of Mental Disorders (DSM-5-TR) of American Psychiatric Association* (2022) [56] and in the *International Classification of Diseases (ICD-10) of the World Health Organization* (2019) [57]. In the *DSM-5-TR*, specifiers are used to discriminate between primary and secondary (resulting from other clinical or neurological conditions) forms of psychiatric disorders [56].

There are very few studies about the prevalence of secondary mental illnesses relevant for the estimation of IEMs incidence, likely due to methodological difficulties, in particular in obtaining sufficiently sized samples [3]. Indeed, the literature on psychiatric manifestations of IEMs consists of case reports or case series of patients with specific disorders, as well as synthetic reviews on the topic.

### 4.2. Type of Onset

In order to distinguish important red flags and suggest timely analysis and investigations, we have classified IEMs in pediatric patients according to their type of psychiatric onset, specifically in acute and chronic categories.

As will be described in the following discussion, acute presentation with episodes of consciousness alteration, behavioral changes, and acute psychotic breaks are characteristic of porphyrias, homocysteine remethylation defects, and urea cycle disorders. Meanwhile, chronic presentation with recurrent psychotic breaks, disorganized behavior, mood and personality changes, and anxiety syndromes are characteristic of cystathionine β-synthase deficiency, Wilson’s disease, cerebrotendinous xanthomatosis, and lysosomal storage diseases. The type of presentation (acute or chronic) can direct one towards the possible metabolic disorders that need to be investigated.

Acute Breaks. Acute mental illness might be the first clinical symptom of an underlying metabolic condition in patients with a completely negative medical history [37]. Symptoms characterizing acute breaks include episodes of mental confusion, delirium, hysteria, vertigo, anxiety, aggressiveness, bizarre behavior, agitation, delusions, schizophrenic behavior, overt psychosis, and, finally, coma [37,40,48].

Acute psychiatric breaks are distinctively associated with acute intermittent porphyria (AIP) or hereditary coproporphyria (HC). Up to half of all patients with these conditions show psychiatric symptoms, 50% of which are psychotic episodes, but also anxiety, depression, and delirium could be the main clinical manifestations of these disorders [37,48]. Acute psychiatric symptoms could also be the only clinical manifestation of these conditions [37]. AIP symptoms are often misdiagnosed mostly as schizophrenia or histrionic personality disorders; moreover, their misdiagnosis as somatoform disorders could be possible because of the presence of recurrent abdominal symptoms during acute distress periods [24,37,40,48]. In the clinical hypothesis of an acute porphyria attack, the diagnostic gold standard is represented by the evaluation of high levels of urinary excretion of porphobilinogen (PBG), performed by Hoesch test and Watson–Schwartz test [58].

Particularly relevant are also hyperammonemia acute breaks, found in urea cycle disorders [e.g., carbamoyl phosphate synthetase deficiency, argininosuccinate synthetase deficiency, ornithine transcarboxylase deficiency (OTC), and organic aciduria]. These are mostly treatable conditions, especially late-onset ones; however, if metabolic investigations are not conducted properly, these conditions can progress to a permanent disability and premature death [49,59]. Ammonia levels must always be determined in clinical pictures of acute encephalopathy accompanied by psychiatric symptoms. Acute breaks can be characterized by delirium-like symptoms, with a confused state and incoherent speech [49].

Chronic Psychiatric Conditions. Subacute or chronic psychiatric manifestations are reported in patients affected by methylenetetrahydrofolate reductase (MTHFR) deficiency, sometimes misdiagnosed as schizophrenia or psychosis [31,36]. Similar psychiatric symptoms are found as well in cobalamin metabolism defects (CblC) [26,46] in creatine transporter defects [53]. Moreover, it has been described that personality disorders, behavioral changes, depression, and obsessive compulsive disorders are the most common psychiatric findings in cystathionine β-synthase deficiency [17]. Psychiatric symptoms, including schizophreniform attacks, episodes of psychomotor agitation, attention deficit hyperactivity disorder, and cognitive impairment, have been reported in cerebrotendinous xanthomatosis and Niemann–Pick type C disease [22,30,47,55].

Depression, generalized anxiety disorder, attention deficit hyperactivity disorder, tic disorder, obsessive compulsive disorder, oppositional defiant disorder, and schizophrenia were also found in patients with Gaucher disease (the most common lysosomal storage disorder) [52], as well as in patients with congenital disorders of glycosylation [20].

It is estimated that half of patients affected by Wilson’s disease (WD) suffer from psychiatric disorders [27]. Psychiatric symptoms may occur in WD in the early stages of development [28] and consist of mood disorders with both depressive and manic manifestations [51], personality changes with irritability and aggressive behavior, schizophrenia-like presentation with hallucinatory symptoms, or even severe episodes of catatonia [27,38].

In Table 2, we report further clinical information concerning the time of onset of these challenging symptoms.

### 4.3. Psychiatric Phenomenology

A wide range of psychiatric symptoms has been reported in association with IEMs. Mood and anxiety disorders are frequently described as clinical manifestations responsive to the underlying chronic disease, while schizophrenic-spectrum disorders, catatonia, eating disorders, and self-injurious behaviors (SIBs) are included among the symptoms characterizing the clinical picture of IEMs as distinctive clinical features of the different disorders. These will be addressed specifically throughout this discussion, presenting key aspects for each metabolic disease. Table 3 illustrates the psychiatric phenomenology of IEMs in pediatric age.

#### 4.3.1. Neurometabolic Diseases and Mood and Anxiety Disorders

Mood and anxiety disorders have been reported in association with IEMs, in particular mood changes (depression with insomnia, social withdrawal, appetitive changes, anergia and apathy, mood instability, and mania), behavioral and personality changes (irritability, aggression, impairments in judgment and insight, and inappropriate behaviors), anxiety syndromes, obsessive compulsive disorder, and chronic attentional deficits, with or without hyperactivity [3].

Mood, anxiety, obsessive compulsive, and attention disorders are described in phenylketonuria [29,42], tetrahydrobiopterin (BH4) deficiencies [41,44], and L-2 hydroxyglutaric aciduria [34].

Depressive disorders have been reported in adolescents with a low folate status [60].

Mitochondrial disorders (MDs) can also be associated with mood and anxiety manifestations. Disturbed mitochondrial function has been suggested to underlie symptoms of bipolar disorder, depressive disorders, and schizophrenia, as described by Al-Owain and colleagues in cases of mitochondrial disorders due to *BCS1L* gene mutation, which manifested hyperactivity in childhood and hypomania in adolescence, later evolving into intermittent psychosis. Anxiety and emotional disturbance were also described [18].

Some inherited neurometabolic diseases that may present with these manifestations in children and adolescents will be covered in the following discussion.

Phenylketonuria

Phenylketonuria (PKU) is a treatable hereditary metabolic cause of intellectual disability linked to a defect in the phenylalanine-hydroxylase (PAH) enzyme that converts phenylalanine (Phe) into tyrosine (Tyr), which leads to Phe accumulation in the blood and body tissues. High Phe levels have been shown to be associated with an increase in neuropsychiatric symptoms, including mood, anxiety, and attention problems. Erlich (2019) described the case of a 15-year-old girl with anxiety and feelings of worry, fatigue, and weight loss. The girl reported difficulty in complying with dietary protein restriction and presented an inadequate metabolic control (blood Phe levels over 500 mmol/L) [29].

At the same time, despite favorable clinical outcomes of early-treated patients compared to untreated or late-treated patients, a lower-than-expected intellectual quotient (IQ) scoring was reported for these patients together with neuropsychological and psychiatric problems [61]. Early-treated children and adolescents with PKU most often show a higher incidence of anxiety, depressive symptoms, social withdrawal, attention problems, and a lower self-esteem [42,50,54].

Executive function (EF) deficits and internalizing symptoms have also been found to coexist in children affected by PKU [25]. EFs are necessary for the management of cognitive, emotional, and behavioral functions [62], as well as for the regulation of inhibition, working-memory cognitive flexibility [63,64,65], attention [66], problem solving, and planning [67,68].

In a study performed by Trimarco et al. (2020), patients affected by autism-spectrum disorders (ASD) were compared with early-treated patients with PKU (age 7–14 years old), and it was shown that the patients with PKU reported more extended damage to their EFs compared to the patients with ASD, especially in the two fundamental domains of inhibition and cognitive flexibility [69].

Another interesting study performed by Manti et al. (2016) investigated psychiatric and neuropsychological aspects in a young adult population (aged 12 to 44 years) of early-treated patients with PKU. More than one-third of the patients showed to meet the criteria for a psychiatric diagnosis (mostly anxiety disorders). Interestingly, psychiatric symptoms’ onset was not related to metabolic control of the disease, but the patients with good metabolic control (Phe levels ≤ 500 μM) in the first 11 years of life showed to have a higher incidence of psychiatric diagnoses [42]. It appears that the psychological burden of the disease and the dietetic treatment act as stressors for patients and their families, as reported also in other chronic diseases of young people, such as diabetes mellitus type I [42,54].

Tetrahydrobiopterin deficiencies

Tetrahydrobiopterin (BH4) deficiencies are a rare group of neurometabolic disorders due to a deficiency in the enzymes involved in BH4 biosynthesis and regeneration [70]. BH4 is the cofactor involved in the metabolism of the amino acids phenylalanine and tryptophan. The enzymes GTP cyclohydrolase I (GTPCH) and 6-pyruvoyl-tetrahydropterin synthase (PTPS) are implicated in the biosynthetic pathway, together with sepiapterin reductase (SR) [71]. Despite differences in biochemical properties, similar clinical features with varying severity may be observed: hyperphenylalaninemia, depletion of brain serotonin and dopamine, and neurodevelopmental disorders [71,72,73].

6-Pyruvoyl-tetrahydropterin synthase deficiency (PTPSd)’s most common clinical presentations are epilepsy, movement disorders, and neurodevelopmental disorders such as global developmental delay and intellectual disability [41].

A study by Manti et al. (2020) evaluated the long-term clinical outcomes of child, adolescent, and adult patients affected by PTPSd and the potential factors able to influence or predict it. The authors found that about half of the patients with PTPSd met the criteria for a psychiatric disorder, including generalized anxiety disorder, depressive disorder, obsessive compulsive disorder, and attention deficit hyperactivity disorder. In addition, they reported an association between IQ scoring, EFs, and adaptive behaviors with the level of Homovanillic acid (HVA) and Phe at birth (lower cerebrospinal fluid (CSF) HVA levels were correlated with more severe scores). Thus, the severity of brain biogenic amine depletion at the time of diagnosis could represent a predictor of neurological and psychiatric outcomes [41].

Pan et al. reported an illustrative case of a patient with severe major depressive disorder and suicidal ideation refractory to treatment [44]. At the age of 14, he presented a first attempt at suicide and, subsequently, a second episode at the age of 15. The patient showed no clinical improvement after initiating therapy with serotonin-norepinephrine reuptake inhibitors (SNRI), antipsychotics, mood stabilizers, and electroconvulsive therapy (ECT). At age 17, after a week of remission, he presented with a new aborted suicide attempt. The boy presented a CSF metabolite profile compatible with GTPCH deficiency. He began therapy with sapropterin, and a progressive improvement in his mood was evident after a month.

#### 4.3.2. Neurometabolic Diseases and Schizophrenia-Spectrum Disorders

Schizophrenia affects about 0.3–0.7 percent of people at some point in their lives [74].

Schizophrenia-spectrum disorders are associated with a wide variety of diseases, including neurometabolic ones. According to the literature, 6% of secondary forms of psychosis have been related to neurometabolic disorders [75]. Presentation of psychotic symptoms may occur years prior to the onset of neurologic symptoms [76].

In *DSM-5-TR* [56], psychiatric diseases due to organic conditions have a distinct section and criteria. Particularly, visual hallucinations (in urea cycle disorders, homocysteine metabolism disorders, and Niemann–Pick disease) and delusions (porphyrias) are highlighted among these criteria [76]. Red flags for secondary IEM causes of juvenile psychosis could be represented by specific associated symptoms: for example, in porphyria, with recurrent abdominal pain and peripheral neuropathy; in Wilson’s disease, with liver dysfunction, jaundice, and splenomegaly; and in Niemann–Pick disease, with an ataxic gait and supranuclear gaze palsy [77]. The early diagnosis of IEMs as secondary causes of psychosis may lead to the appropriate treatment of these clinical features in which a high sensitivity to antipsychotics and a treatment resistance have been described, whereas simple dietary modifications or replacements may result in a more resolute and appropriate treatment [76].

Table 4 summarizes inherited neurometabolic diseases that may present with schizophrenia-spectrum disorders in children and adolescents. They will be presented individually in the following discussion.

Porphyrias

Porphyrias are a group of eight inherited metabolic disorders of heme biosynthesis [78]. The type of porphyria and the associated clinical manifestations depend on several biosynthesis step blocks, leading to the aggregation of porphyrin or its precursors [delta-aminolevulinic acid (ALA) and porphobilinogen (PBG)] and causing characteristic clinical features: acute neurovisceral attacks, skin lesions, or both. Clinical signs can appear in childhood, although they usually appear in adults [79]. Acute psychiatric breaks are distinctively associated with acute intermittent porphyria (AIP) or hereditary coproporphyria (HC). In acute hepatic forms, psychiatric symptoms can be associated with severe abdominal pain, nausea, and emesis [76,80].

The most frequent psychiatric symptoms include mood disturbances, anxiety, behavioral and sleep disturbances, and psychotic features, such as hallucinations, course-of-thought disorders, and delusion states [37,48].

Triggers of acute forms may be the use of several P450-inducing drugs, hormones (progesterone), nutritional factors (reduced intracellular glucose), stress, and alcohol [79]. The diagnosis is based on urine testing for excess PBG and measuring ALA in the blood. Treatment consists of the injection of human hemin and carbohydrates’ infusion [78].

Some interesting cases of psychiatric symptoms in children and adolescents are described in the literature. Santosh and Malhotra (1994) reported a case of a 14-year-old boy affected by AIP, emphasizing the possible role of triggers (such as pharmacological triggers or others) in acute and intermittent manifestation of these diseases. This boy experienced six episodes of an acute onset of psychiatric symptoms, characterized by a variety of manifestations, ranging from disorientation, muteness, echolalia, delusions, hallucinations, hypomania, and even catatonia [48].

Kumar reported a case of a 15-year-old girl with an acute porphyric attack manifesting exclusively as psychosis without any accompanying somatic features. The girl experienced bizarre behavior, loose and tangential speech, and a non-linear thought process with paranoid content [37].

Mandoki and Sumner (1994) reported a case of a 9-year-old female with coproporphyria. The girl appeared dysphoric, emotionally labile, and aggressive and reported seeing and hearing ghosts, sleep disturbance, stomachaches, and headaches [40].

Cerebrotendinous Xanthomatosis

Cerebrotendinous xanthomatosis (CTX) is a disease that affects bile acid synthesis and is caused by an autosomal recessive mutation in CYP27A1. It induces disturbances in the mitochondrial enzyme sterol 27-hydroxylase, involved in the synthesis of cholic and chenodeoxycholic acids, leading to an accumulation of cholesterol and cholestanol in the brain and tissues, particularly tendons [81].

Based on the different ages of onset, CTX is characterized by diarrhea, cataract, tendon xanthomas, and progressive neurological dysfunction and psychiatric disturbances [5,81].

Psychiatric symptoms have been reported in CTX, including schizophreniform attacks, episodes of psychomotor agitation, ADHD symptoms, and cognitive impairment [22,30].

Most psychiatric symptoms that occur during childhood or adolescence are nonspecific, including behavior and personality disorders, learning disabilities, or intellectual disabilities [22,30].

The diagnosis is based on clinical signs and high plasmatic and tissue concentrations of cholestanol. The treatment consists of chenodeoxycholic acid [81].

Niemann–Pick type C Disease

Niemann–Pick type C disease (NPC) is an autosomal recessively inherited lysosomal disorder. Mutations in the *NPC1* or *NPC2* gene produce the defective traffic of endocytosed LDL cholesterol, glycosphingolipids, and sphingosine, leading to their abnormal storage inside the lysosomal compartment. Accumulation of these products can be found in the liver, spleen, and brain. The clinical presentation of this disease is extremely varied, and the manifestations may be primarily hepatic, neurological, and psychiatric [82].

The classic presentation of this disease occurs during childhood, with progressive cognitive and neurological deterioration, initially characterized by developmental coordination disorder, ataxia, and vertical supranuclear gaze palsy. Dysarthria, dysfunctional dysphagia, and deafness may coexist [82]. Psychosis is a common presentation of adolescent-onset NPC [5,23].

The diagnosis is made by molecular genetic testing (pathogenetic variants in NPC1 or NPC2) and a skin biopsy with a Filipin staining test and a fibroblast culture, even though, more recently, the diagnosis of this disease has become easier with blood oxysterols doses, which are elevated in most of the patients [82]. The treatment is based on Miglustat, which has been seen to slow the progression of the disease [83].

Bonnot and colleagues (2019) have shown some cases of NPC exhibiting schizophrenia-like disorders with neurological signs [83], and Wouters et al. (2014) reported the case of a 16-year-old girl who presented with an abrupt psychotic symptomatology (auditory and visual hallucinations, paranoid delusions) and a medical history of visceral and neurological dysfunction [55]. In addition, Giannitelli et al. (2018) and Sandu et al. (2009) reported cases of children and adolescents with Niemann–Pick type C, who initially received psychiatric diagnoses in the schizophrenia spectrum [33].

It should be important for researchers to pay attention to cases of a suspected diagnosis of ‘hebephrenic schizophrenia’ (also called ‘disorganized schizophrenia’), which includes changes in affect, drive, and thought disorders (World Health Organization, 2019).

Disorders of the Homocysteine Metabolism

High levels of homocysteine are toxic to neurons and blood vessels and can induce DNA strand breakage, oxidative stress, and apoptosis. The homocysteine–methionine cycle can be affected by several genetic mutations, diet, kidney and gastrointestinal diseases, and drugs. The associated disorders are related to the absence of distinct enzymes.

Cystathionine β-synthase deficiency—homocystinuria—is an autosomal recessive condition. Cystathionine β-synthase converts homocysteine into cystathionine with the help of cofactors, including vitamins B12 and B6 and folic acid. Homocysteine accumulation is responsible for highly variable clinical features with multisystemic involvement (eyes, skeleton, nervous system, and vascular system). Childhood onset can be characterized by a severe multisystemic disease or remain asymptomatic until adulthood [84]. Kevere et al. reported the association between high homocysteine levels and schizophrenia in children and adolescents [36]. Similarly, Hidalgo Mazzei et al. (2014) described the case of a 17-year-old boy presenting with a first episode of psychosis and unknown homocystinuria due to cystathionine β-synthase deficiency, which led to a lethal outcome [35]. In addition, Abbott and colleagues showed that personality disorders, behavioral disturbances (such as aggressiveness), depression, and obsessive compulsive disorders are very common psychiatric findings [17].

MTHFR (methylenetetrahydrofolate reductase) deficiency-related hyperhomocysteinemia is an autosomal recessive defect caused by a mutation in the *MTHFR* gene. It can show variable expression and a wide clinical presentation, ranging from asymptomatic pictures to neurological signs and psychiatric disorders. Morris and colleagues detected a low folate status in depressed adolescents [60]. In addition, a study by Kevere et al. showed that high homocysteine levels and the MTHFR genotype are risk factors for affective disorders and schizophrenia in children and adolescents [36]. This association was also described by Freeman et al. (1975) through the case of a 15-year-old girl with recurrent episodes of mental deterioration and schizophrenia-like behavior responsive to the administration of folic acid. The girl had MTHFR deficiency-related homocystinuria [31].

Deficiency in CblC (cobalamin C) is an inborn metabolic disorder that causes combined methylmalonic acidemia and homocystinuria, with an impaired intracellular synthesis of the active forms of vitamin B12 (cobalamin), which results in increased levels of methylmalonic acid and homocysteine in the blood and urine [85].

This disease typically presents with growth difficulties, acute neurological deterioration, cognitive impairment, lethargy, epilepsy, microcephaly, “salt and pepper” type retinopathy, and signs of megaloblastic anemia (pallor, fatigue, and anorexia) [86].

Roze and colleagues have described the case of a 16-year-old girl initially presenting with a psychosis characterized by a dissociative state, an altered course of thought, and visual and auditory hallucinations and, subsequently, with a severe progressive neuropathy requiring mechanical ventilatory support. The girl also had a family history of cblC deficiency, in her 24-year-old sister [46].

Chen and colleagues (2022) described patients with late-onset cblC deficiency (age of onset from 10 to 20 years), more than three-quarters of whom presented neuropsychiatric diseases, including psychotic behavioral disorders (short temper, speaking nonsense words, hallucination, apathy, and overeating), mental regression, memory loss, study weariness, and decrease in grades [26].

Urea Cycle Disorders

Urea cycle defects (UCDs) are among the most common IEMs. They can arise at any age and result in deficiency in one of the six enzymes involved in the urea cycle, whose role is to remove ammonia from the bloodstream by converting nitrogen into urea. High ammonia levels result in toxicity for the brain [87].

The severity of these disorders is related to the extent of the enzyme deficiency. There could be mild forms that generally show nausea, vomiting, and headache in patients exposed to a high protein intake, as well as acute neurological and psychiatric pictures [87]. Onset may occur in the neonatal period, in childhood, or in adolescence/early adulthood.

Psychiatric signs are frequent and may consist of mood disorders or auditory or visual episodic hallucinations. The course of these disorders may be acute or subacute [49].

Acute breaks can be characterized by delirium-like symptoms, with a confused state and incoherent speech [49].

The combination of an acute picture of visual hallucinations and vomiting in the context of taking medication or a high-protein regimen should indicate an underlying urea cycle disorder (for example, in people consuming high-protein supplements). Emergency ammonia assays should be part of the basic investigations conducted on all patients with encephalopathy, at all ages [49,59].

Diagnosis confirmation is obtained by the determination of the ammonia blood levels and molecular genetic testing. It is important to make an early diagnosis because of the risk of fatal outcomes, worsening neurological signs (seizures), and encephalopathy, leading to lethargy and coma [49,59].

Acute treatment of these disorders consists of sodium-benzoate or sodium-phenylbutyrate and high amounts of intravenous glucose and lipids. Hemodialysis and hemodiafiltration are the most efficient treatment strategies for plasmatic ammonia reduction [59]. Chronic treatment consists of dietary protein restriction [59].

Wilson’s Disease

Wilson’s disease (WD) is an autosomal recessive condition caused by a mutation in the ATP7B gene, encoding for a copper transporter protein (P-type ATPase), which leads to copper accumulation in the liver, kidneys, bones, brain, and eyes. In WD, liver damage appears in the second decade of life, followed by neurological and psychiatric disorders in the third decade [88].

It is estimated that half of patients with WD suffer from psychiatric disorders and that about 20% of them have no organic symptoms [27]. Psychiatric symptoms may occur in the early stages of development [28].

In order of frequency, psychiatric manifestations consist of mood disorders with both depressive and manic manifestations [51], personality changes with irritability and aggressive behavior, and schizophrenia-like presentation with hallucinatory symptoms or even severe episodes of catatonia [27,38]. It is likely that diagnosis in patients with a predominantly neuropsychiatric presentation could be delayed from the symptoms’ onset, leading to a worse outcome compared to patients with hepatic symptoms [27]. Alam et al. (2012) described a case of a 15-year-old boy whose disease onset was preceded by behavioral disorders such as excessive anger, occasional irrelevant speech, and concentration deficit [19].

The cognitive deficits that occur in patients with WD are secondary to metabolic changes and are potentially reversible when an anti-copper treatment is performed. MRI abnormalities such as cortico–striatal pathway lesions could be associated with cognitive deficits [89].

The diagnosis is achieved by MRI, which reveals hyperdense signals in the thalamus and basal ganglia, a serum ceruloplasmin test, a 24 h urine copper test, a liver biopsy for histology, histochemistry, and copper quantification, genetic testing, or detection of the classic Kayser–Fleischer ring by slit lamp examination [88].

The treatment includes avoidance of copper-rich foods and assumption of penicillamine and trientine that reduce copper absorption by zinc and copper chelation [90].

Selective serotonin reuptake inhibitors (SSRIs), as in Parkinson disease or Huntington disease, have been successfully used as a first-line treatment of depressive syndromes in WD [89]. The treatment of psychotic disorders turns out to be a great challenge in these patients, since the use of neuroleptics can exacerbate their psychiatric symptoms and increase the risk of developing neurological adverse effects [89]. Therefore, agents with a low risk of extrapyramidal symptoms as well as a low hepatic risk are recommended as the first-line treatment for psychosis in WD patients [89]. Electroconvulsive therapy can be effective in severe symptoms or neuroleptic side effects [91].

#### 4.3.3. Neurometabolic Diseases and Catatonia

Catatonia is one of the most severe psychiatric syndromes in young people and is associated with a high proportion of organic diseases that should always be excluded [92].

Catatonia is a syndrome characterized by the association of motor abnormalities and psychic symptoms [93], generally presenting with a typical neurological examination [94]. The symptoms are often considered functional and must be understood at the level of the subjects’ experience [92].

Several varieties can be distinguished [93]: stuporous catatonia, excited catatonia, malignant catatonia, and psychomotor automatism.

According to Cornic et al. (2009), they can be a consequence of differential motor responses to hallucinations and/or delusions that lead to intentionality dysfunction (e.g., when a patient resists hallucinatory orders to commit suicide), behavioral pattern-planning dysfunction (e.g., when a patient strictly adheres to voices or hallucinations which order movements, so-called “De Clérambault’s psychomotor automatism” [95]), or an emotional regulation dysfunction characterized by extreme emotional involvement (e.g., in stuporous anxious states).

Regardless of psychiatric presentation, organic conditions can be associated in 20% of cases [38]. This last observation was recognized by the *DSM-5-TR* [56] under the title of “catatonic disorder due to a general medical condition”. Associated medical conditions are infectious diseases, genetic conditions, neurological diseases, intoxications, and metabolic conditions [94].

Many inborn errors of the metabolism (IEM) can present with a catatonic symptomatology such as, for example, urea cycle defects, homocysteine remethylation defects, and porphyrias, presenting with acute attacks of confusion. Homocystinuria, Wilson’s disease, cerebrotendinous xanthomatosis, and lysosomal storage diseases may be the cause of isolated psychiatric manifestations developing in adolescence or in a previously typical patient [38]. Santosh and Malhotra (1994) reported a case of a 14-year-old boy affected by AIP who experienced six episodes of an acute onset of psychiatric symptoms, characterized by a variety of manifestations, including catatonia [48]. Catatonic symptomatology exacerbations after treatment are atypical presentations characteristic of IEMs [38].

The treatment approach used in youth is similar to the one used in adult catatonia and includes the use of high doses of benzodiazepines, such as lorazepam, or other sedative drugs and anti-Parkinsonism drugs (e.g., amantadine) [94].

#### 4.3.4. Neurometabolic Diseases and Eating Disorders

According to *DSM-5-TR* [56], eating and nutrition disorders are defined by eating-related behaviors that result in impaired food consumption or absorption and significantly affect physical health or psychosocial functioning. They include pica, rumination disorder, avoidant/restrictive eating disorder, anorexia nervosa, bulimia nervosa, binge-eating disorder, and eating disorders with other specification or without specification [56].

In the literature, several studies concerning eating habits in patients with metabolic disorders show that the most frequent symptom is food restriction mimicking anorexia nervosa and food selectivity. Gardeitchik et al. (2012) hypothesize that protein aversion may be a diagnostic clue in patients presenting with refusal to eat, frequent emesis, behavioral issues, intellectual disability, and impaired consciousness episodes [32].

Confirming this hypothesis, Serrano and colleagues reported that patients with late-onset urea cycle disorders initially displayed eating disorders as part of their neuropsychiatric symptoms [49]. In some cases, recurrent vomiting and refusal of protein-rich food were observed. One of these cases was a 7-year-old boy with carbamoyl phosphate synthetase deficiency who spontaneously refused protein-rich foods at 4 years of age. In addition, a 3-year-old patient with argininosuccinate synthetase deficiency showed refusal of protein-rich food and pica (he consumed cloth, paper, and other materials). After treatment with carnitine and arginine and a diet which restricted protein, he showed an appropriate behavior [49]. Also, Niwinsky et al. (2021) described the case of a 15-year-old girl with a clear protein aversion. The patient was diagnosed with ornithine transcarbamylase (OTC) deficiency (hyperammonemia type II), following an initial diagnosis of pervasive developmental disorder, selective mutism, and anorexia nervosa [43]. Therefore, as these symptoms are very common in patients with urea cycle disorders, they should alert physicians to diagnose and treat them as soon as possible.

Methylmalonic Acidemia (MMA) and Propionic Acidemia (PA)

Methylmalonic acidemia (MMA) and propionic acidemia (PA) are congenital disorders of the branched-chain amino acid metabolism. MMA is a disorder of the methylmalonate and cobalamin metabolism. Two main forms of this disease have been identified: isolated and combined methylmalonic aciduria, and homocystinuria, caused by various specific genetic mutations [96]. PA is caused by a deficiency in propionyl-CoA carboxylase (PCC), the enzyme which catalyzes the conversion of propionyl-CoA into methylmalonyl-CoA.

The spectrum of acidemia varies from neonatal onset (most common form), with severe progressive encephalopathy, to a late onset form, with hyperammonemia and hyperlactatemia, or a more insidious onset, with the development of complications. Manifestations over time may include growth failure, intellectual disability, seizures, basal ganglia injury, pancreatitis, cardiomyopathy, and chronic renal failure [97].

Therapy is based on a low-protein diet, limiting amino acids’ precursors, and the addition of special mixtures of essential amino acids to maintain typical growth and nutrition. Liver transplantation has been proposed as a treatment modality to reduce metabolic decompensations that are not controlled by medical management [98].

Several cases of MMA and PA with feeding disorders were described by Touati et al. (2006). All the patients in their study were treated following the same enteral feeding protocol with a low-protein diet adapted to individual tolerance, carnitine, antibiotics, and only the occasional use of a blend of amino acids [99].

Rahmandar et al. (2014) described an adolescent patient with cblC deficiency who displayed a late-onset phenotype associated with an altered mental status and anorexia, which resolved after treatment [45].

Mitochondrial Neurogastrointestinal Encephalomyopathy

Mitochondrial neurogastrointestinal encephalomyopathy (MNGIE) is a rare, fatal, autosomal recessive disease associated with a deficiency in the thymidine phosphorylase (TP) enzyme, encoded by the TYMP gene [100]. This mutation causes the accumulation of thymidine and deoxyuridine and secondary alterations in the mitochondrial DNA (multiple deletions and depletion) [101]. The average age of onset is estimated to be around 19 years (ranging from 5 to 50 years), with a progressive course of disease, a poor prognosis, and an average age of death of 37 years [102]. The clinical features include peripheral neuropathy (more evidently affecting the lower limbs), ophthalmoparesis, gastrointestinal dysmotility (nausea, dysphagia, gastroesophageal reflux, postprandial vomiting, episodic abdominal pain, and diarrhea), cachexia, and diffuse leukoencephalopathy [103,104]. The diagnosis of MNGIE can be established through the detection of biallelic pathogenic variants in TYMP, significantly reduced levels of thymidine phosphorylase enzyme activity, or elevated plasma concentrations of thymidine and deoxyuridine [101].

MNGIE can mimic the symptoms of different pathologies, such as anorexia nervosa, gastrointestinal diseases, neuropathies, defects of oxidative phosphorylation, and Leukodystrophies [102].

The current therapeutic strategies for MNGIE are hemodialysis, the replacement of the deficient enzyme by platelet transfusions, and the replacement of the missing enzyme by hematopoietic stem cell transplantation [100,105].

Libernini et al. (2012) have analyzed the cases of two brothers with a new mutation (compound heterozygous) in the thymidine phosphorylase (TYMP) gene, associated with clinically different presentation and progression. The first patient was hospitalized at the age of 16 with a suspected diagnosis of anorexia nervosa. In his 13-year-old brother, the clinical signs were a sensorineural hearing deficit and muscular hypotrophy. The older brother died at the age of 17 from acute pulmonary edema after a worsening of his general conditions, despite two attempts of platelets transfusion. The younger brother, instead, at the age of 15 was enrolled in an allogeneic hematopoietic stem cell transplant program [39].

Hematopoietic stem cell transplantation has the potential to restore enzyme activity and induce the normalization of toxic nucleoside concentration [106]. Therefore, it is important to recognize patients with MNGIE early, because early hematopoietic stem cell transplantation could be a lifesaver.

#### 4.3.5. Neurometabolic Diseases and Self-Injurious Behaviors

Self-injurious behaviors (SIBs) are common clinical conditions affecting a wide group of patients. Severe SIBs can be devastating and potentially life-threatening [107]. “Self-harm” means damage to one’s body through direct self-inflicted injury (any kind of damage to a body surface causing bleeding, bruising, or pain), which leads to only minor or moderate physical damage in the absence of suicidal intent or sexual arousal [107]. Particularly common in the psychiatric population and in patients with varying degrees of intellectual disability, it can also occur after exposure to psychotropic substances or during psychotic episodes, as well as in patients with rigid/repetitive behavioral patterns or an enhancement in their pathological behavioral output [107,108,109]. Theories regarding pathophysiological implications refer to abnormalities of dopaminergic neurotransmission that may represent a risk factor for SIBs, due to dopamine’s involvement in repetitive behaviors [110]. These neurochemical changes suggest a role in the genesis of these basal ganglia disorders [111,112] with subsequent feedback on SIBs therapies.

Of all neurometabolic disorders, Lesch–Nyhan syndrome is the most frequently associated with SIBs, followed by 6-pyruvoyl-tetrahydropterin synthase deficiency, late-treated phenylketonuria (PKU), and hepatolenticular degeneration [110].

Lesch–Nyhan Syndrome

Lesch–Nyhan syndrome (LNS) is an inborn X-linked recessive disorder caused by a deficiency in the hypoxanthine-guanine phosphoribosyltransferase (HGPRT) enzyme, involved in the purine salvage pathway. This defect results in the accumulation of purine nucleotides and uric acid. Male patients are usually affected, while female patients are heterozygous carriers (usually asymptomatic) [113].

This condition is characterized by hyperuricemia, neurodevelopmental delay, involuntary movements, generalized dystonia, and self-injurious behaviors [114,115]. A wide spectrum of clinical manifestation severity is recognized, based on the extent of HGPRT deficiency [116,117,118].

Self-injurious behavior in this condition has been described by Anderson and Ernst (1994) in several cases. The most common type was biting some part of the body (lips, cheeks, or fingers), followed by throwing an arm, leg, or head out, arching the spine, snapping back the head, and head banging [21].

In LNS, there is evidence of the selective dysfunction of dopaminergic pathways, with dopamine reduction in the basal ganglia. A functional loss of 60–90% of the nigrostriatal and mesolimbic dopaminergic terminals has been described [110,119]. Low levels of dopamine-synthesizing enzymes have been found in the caudate nucleus, in the putamen, and in the nucleus accumbens [120].

Diagnosis is suspected in the presence of psychomotor delay and elevated uric acid levels in the blood and urine and can be confirmed by the determination of HPRT enzymatic activity and a molecular genetic analysis [121]. The treatment includes monitoring hyperuricemia levels by allopurinol administration. In addition, self-mutilation requires physical restrictions and behavioral and drug therapies (benzodiazepine and carbamazepine) [121], and deep-brain stimulation (DBS) for SIB treatment targets the basal ganglia, including the internal pale (GPi) and the nucleus accumbens [122].

## 5. Discussion

In this systematic review, we provided a comprehensive analysis of different psychiatric manifestations in pediatric patients with IEMs. The results of this study were reported according to two criteria, by type of symptoms’ onset during pediatric age and by possible psychiatric features related to IEMs.

First of all, we classified IEMs in pediatric patients according to their type of psychiatric onset, specifically in acute and chronic categories.

Acute mental illness might be the first clinical symptom of an underlying metabolic condition in patients with a completely negative medical history [37]. Symptoms characterizing acute breaks include episodes of mental confusion, delirium, hysteria, vertigo, anxiety, aggressiveness, bizarre behavior, agitation, delusions, schizophrenic behavior, and overt psychosis. These manifestations have been described in porphyrias, homocysteine remethylation defects, and urea cycle disorders [37,40,48].

Symptoms characterizing chronic presentation include recurrent psychotic breaks, disorganized behavior, mood and personality changes, and anxiety syndromes. These manifestations have been described in cystathionine β-synthase deficiency, Wilson’s disease, cerebrotendinous xanthomatosis, Nieman–Pick type C, lysosomal storage diseases, and congenital disorders of glycosylation [17,20,22,26,30,31,36,46,47,52,55].

These patients may be misdiagnosed and not receive adequate treatment [4,6]. Therefore, the type of presentation (acute or chronic) can direct professionals towards the possible metabolic disorders that need to be investigated. Standard diagnostic procedures for individual IEMs often involve several tests and can be time-consuming and expensive, resulting in diagnostic delays. Next-generation sequencing (NGS), however, can be used as a noninvasive method, with high costs, to screen for genes associated with treatable IEMs with psychotic symptoms [123].

Secondly, we grouped psychiatric phenomenology according to five main clinical manifestations: mood and anxiety disorders; schizophrenia-spectrum disorders; catatonia; eating disorders; and SIBs. This categorization was provided with the aim of being supportive for clinicians, in order to distinguish different clinical manifestations which raise suspicion of an underlying IEM. Psychiatric symptoms may manifest with specific associated symptoms: such as in porphyria, with recurrent abdominal pain and peripheral neuropathy; in Wilson’s disease, with liver dysfunction, jaundice, and splenomegaly; or in Niemann–Pick disease; with an ataxic gait and supranuclear gaze palsy [77]. These red flags may help psychiatrists in this difficult diagnostic process. Moreover, a division by the time of onset of psychiatric manifestations in patients affected by IEMs could be essential in clinical practice among pediatric-age patients. For this reason, we developed a diagnostic chart based on the results of this study, as represented in Table 5.

Mood and anxiety disorders have been considerably reported in IEMs. Mood, anxiety, obsessive compulsive, and attention disorders are described in PKU [29,42], BH4 deficiencies [41,44], and L-2 hydroxyglutaric aciduria [34]. Depressive disorders have been reported in adolescents with a low folate status [60]. These symptoms are also described in mitochondrial disorders [18].

Schizophrenia-spectrum disorders are associated with a wide variety of diseases, including neurometabolic ones.

Psychotic features, such as hallucinations, course-of-thought disorders, and delusion states, are distinctively associated with AIP or to hereditary coproporphyria [37,40,48]. Acute porphyric attacks may manifest exclusively as psychosis without any accompanying somatic features [37] or be associated with severe abdominal pain, nausea, and emesis [40].

Also, CTX may manifest with schizophreniform attacks and episodes of psychomotor agitation [22,30].

Psychosis is a common presentation of adolescent-onset NPC [23]: patients may exhibit schizophrenia-like disorders without neurological signs [23] or with a medical history of visceral and neurological dysfunction [55].

The association between high homocysteine levels and schizophrenia in children and adolescents has been reported [36]; indeed, psychosis could be the first symptom of homocystinuria due to cystathionine b-synthase deficiency [35] or cbl-C deficiency [46]. At the same time, a schizophrenia-like behavior, responsive to the administration of folic acid, has been described in MTHFR deficiency-related homocystinuria [31].

The combination of an acute picture of visual hallucinations and vomiting in the context of taking medications or a high-protein regimen should indicate an underlying urea cycle disorder [49].

It is estimated that half of patients affected by Wilson’s disease suffer from psychiatric disorders [27]. Psychiatric symptoms may occur in the early stages of development [28] and include a schizophrenia-like presentation with hallucinatory symptoms or even severe episodes of catatonia [27,38]. The treatment of psychotic disorders in WD has turned out to be a great challenge in these patients, since the use of neuroleptics can exacerbate their psychiatric symptoms and increase the risk of developing neurological adverse effects [89]. Therefore, agents with a low risk of extrapyramidal symptoms as well as a low hepatic risk are recommended as the first-line treatment for psychosis in WD patients [89].

Many inborn errors of the metabolism (IEMs) can present with a catatonic symptomatology: for example, urea cycle defects, homocysteine remethylation defects, and porphyrias present with acute attacks of confusion. Homocystinuria, Wilson’s disease, cerebrotendinous xanthomatosis, and lysosomal storage diseases may be the cause of isolated psychiatric manifestations developing in adolescence or in a previously typical patient [38].

Eating disorders are well described in IEMs. Protein aversion may be a diagnostic clue in patients presenting with refusal to eat, frequent emesis, and behavioral issues [32]. These symptoms are very common in patients with urea cycle disorders, and they should alert physicians to diagnose and treat them as soon as possible [43,49]. Eating disorders and anorexia nervosa can be characteristic of methylmalonic acidemia [45]. MNGIE can mimic anorexia nervosa [39]. Its manifestations include nausea, dysphagia, gastroesophageal reflux, postprandial vomiting, episodic abdominal pain, diarrhea, and cachexia [102]. MNGIE is a rare and fatal disease; therefore, it is important to recognize these patients early, because early hematopoietic stem cell transplantation could be a lifesaver.

Self-injurious behaviors are common manifestations of a wide group of patients and can be potentially life-threatening [107]. Of all neurometabolic disorders, Lesch–Nyhan syndrome is the most frequently associated with SIBs, followed by 6-pyruvoyl-tetrahydropterin synthase deficiency, late-treated phenylketonuria (PKU), and hepatolenticular degeneration [110]. The most common SIBs types in LNS are biting some parts of the body (lips, cheeks, or fingers), followed by throwing an arm, leg, or head out, arching the spine, snapping back the head, and head banging [21].

### 5.1. Strengths and Limitations

This work presents an up-to-date review of the current literature on psychiatric manifestations of IEMs in pediatric age, considering different types of classification. A strength of this research is the inclusion of a broad range of psychiatric manifestations in children and adolescents with different IEMs; thus, it is not limited to one type of IEM or a specific psychiatric manifestation. This process allowed us to explore in depth the facets of apparently different disorders.

A limitation is the variability in the included studies, characterized by different-sized samples, in most cases patients with specific disorders, and a limited structured assessment of the psychopathology. Moreover, only studies in English were included, which may increase the chance of reporting bias.

### 5.2. Future Directions

The early diagnosis of IEMs is crucial for clinical outcome and treatment. Due to the correlation of IEMs and psychiatric features in the neurodevelopmental age, psychiatric assessments should become a standard part of the diagnostic tools used for suspected IEMs among pediatric-age patients. In addition, future research should focus on broad samples of pediatric patients with different IEMs, including structured assessments of psychopathology.

## 6. Conclusions

Psychiatric disorders in children and adolescents have been reported as features of inherited metabolic disorders (IEMs). Early and sudden psychiatric symptoms could be related to dramatic or even treatable features of IEMs, thus being of critical relevance to clinicians. The early diagnosis of IEMs plays an important role in the clinical outcomes and specific treatment of the patients affected.

In the literature, several clinical attempts have been completed to present a systematic and practical classification of these comorbid symptoms, based on their clinical onset, timing, and course.

Moreover, in this paper, we tried to overview the most recurrent and specific psychiatric features related to IEMs in children and adolescents by type of onset and psychiatric phenomenology. These psychiatric features could be misdiagnosed in clinical practice as a primary diagnosis, with related delays in early and appropriate treatments. Thus, highlighting the possible correlation between IEMs and psychiatric features in the neurodevelopmental age is of extreme importance for clinicians and their young patients. In our opinion, psychiatric assessments should become a standard part of the diagnostic tools used for suspected IEMs in pediatric-age patients. Furthermore, future research should focus on broad samples of pediatric patients with different IEMs, including structured assessments of psychopathology.

## Figures and Tables

**Table 1:** Included articles in this systematic review. AIP = acute intermittent porphyria; cblC = cobalamin C; CBSd = cystathionine β-synthase deficiency; CDG = congenital disorders of glycosylation; CTP = creatine transporter; CTX = cerebrotendinous xanthomatosis; GD = Gaucher disease; GTPCH = guanosine triphosphate-cyclohydrolase; HC = hereditary coproporphyria; HGA = hydroxyglutaric aciduria; IEM = inborn error of the metabolism; LNS = Lesch–Nyhan syndrome; MDs = mitochondrial disorders; MMA = methylmalonic acidemia; MNGIE = mitochondrial neurogastrointestinal encephalomyopathy; MTHFR = methylenetetrahydrofolate reductase; NPC = Niemann–Pick type C disease; OCT = ornithine carbamoyltransferase; PKU = phenylketonuria; PMs = psychiatric manifestations; PTPsd = 6-pyruvoyl-tetrahydropterin synthase deficiency; SIBs = self-injurious behaviors; UCDs = urea cycle defects; and WD = Wilson’s disease.

Study	N. of Patients	Age	IEM	Psychiatric Manifestations	Red Flags/Other Manifestations	Type of Onset of PMs	Time of Onset of PMs
Abbott et al., 1987 [17]	63	19 years (range 8–30 years, SD 11 years)	Homocystinuria due to CBSd	Behavior and personality disorders, depression, obsessive compulsive disorder	/	Chronic	20 years (range 10–31 years, SD 11 years)
Al-Owain et al., 2013 [18]	9	14.9 years (range 7½-28 years)	MDs due to BCS1L gene mutation	Hypomania, anxiety, intermittent psychosis, attention deficit hyperactivity disorder	Lactic acidosis, cortical visual dysfunction	Chronic	/
Alam et al., 2012 [19]	1	15 years	WD	Behavioral disorders, irrelevant speech, attention deficit	Corneal Kayser–Fleischer ring, dystonia, tremor, dysphagia	Chronic	15 years
Albokhari et al., 2022 [20]	7	5.14 years (range 3–9 years)	CDG	Aggressive behavior, oppositional defiant disorder, obsessive compulsive disorder, attention deficit hyperactivity disorder	Hypotonia, protein-losing enteropathy, hepatic involvement, developmental delay, skeletal abnormalities	Chronic	/
Anderson and Ernst, 1994 [21]	40	13.9 years (range 2–32 years)	LNS	SIBs	Developmental delay, teething and renal problems	Chronic	3 years (range 1–10 years, SD 1.96 years)
Bonnot et al., 2010 [22]	2	/	CTX	Aggressive behavior, oppositional defiant disorder learning disabilities, attention deficit hyperactivity disorder	Diarrhea, cataract, pes cavus and hammer toes, progressive neurological dysfunction, cognitive impairment	Chronic	11 years (range 9–13 years)
Bonnot et al., 2019 [23]	44	29.2 years (range 1.9–69.8 years)	NPC	Psychotic breaks, mood and anxiety disorders, impaired impulse control	Ataxia, dysarthria, supranuclear gaze palsy, epilepsy, dysphagia, cataplexy, cognitive impairment, visceral symptoms	Acute and Chronic	17.9 years (range 2.5–67.9 years)
Boon and Ellis, 1989 [24]	2	8.5 years (range 6–11 years)	AIP	Psychotic breaks (auditory and visual hallucinations, bizarre and disorganized thought and behavior)	Abdominal pain, diarrhea, nausea, and emesis	Acute	8.5 years (range 6–11 years)
Cappelletti et al., 2013 [25]	35	11.5 years (range 4–24 years, SD 6.2 years)	PKU	Mood and anxiety disorders	Executive functions deficit	Chronic	/
Chen et al., 2022 [26]	56	12 years (range 10–20 years)	Late-onset cblC deficiency	Psychotic symptoms (hallucinations, bizarre and disorganized thought) and behavioral disorders	Movement disorders, mental regression, cardiovascular diseases, pulmonary hypertension, visual impairments	Acute and Chronic	/
Dening and Berrios 1989 [27]	195	19.7 years (SD 8.7 years)	WD	Irritability, personality changes, aggressive behavior, depression, catatonia	Movement disorders, corneal Kayser–Fleischer ring, hepatic disorders, cognitive impairment	Chronic	/
Dening and Berrios 1990 [28]	129	18.8 years (SD 7.9 years)	WD	Depression, irritability, personality changes, aggressive behavior	Movement disorders, corneal Kayser–Fleischer ring, hepatic disorders, cognitive impairment	Chronic	/
Erlich, 2019 [29]	1	15 years	PKU	Generalized anxiety disorder, depression, history of suicidal ideation	Mild conductive hearing loss, obesity, blonde hair, blue eyes, fair complexion	Chronic	/
Fraidakis, 2013 [30]	6	35 years (range 17–58 years)	CTX	Oppositional defiant behaviors and deficits in attention, impulsivity, aggressiveness, anxiety, depression	Diarrhea, pes cavus and hammer toes, progressive neurological dysfunction, cognitive impairment	Chronic	31.8 years (range 6–50 years)
Freeman et al., 1975 [31]	1	15 years	MTHFR deficiency	Psychotic symptoms (hallucinations, delusions, flat affect), catatonic posturing, withdrawal, anorexia	Tremor, cognitive impairment	Chronic	15 years
Gardeitchik et al., 2012 [32]	90	1.62 years (range 0.11–43.14 years)	UCDs	Confusion, behavioral problems, irritability, protein aversion, food refusal, adverse reactions to high-protein foods	Emesis, lethargy, altered consciousness, developmental delay, failure to thrive, hypotonia	Acute	/
Giannitelli et al. 2018 [33]	160	Childhood and adolescence	NPC, Hunter syndrome	Psychotic symptoms	/	Acute and chronic	Childhood and adolescence
Gökçen et al., 2013 [34]	1	13 years	L-2 HGA	Anxiety	Learning disorders, intentional tremors, truncal ataxia, titubation, dysmetria, dysdiadochokinesia, dysarthria	Acute	13 years
Hidalgo Mazzei et al., 2014 [35]	1	17 years	Homocystinuria due to CBSd	Psychotic symptoms (hallucinations, delusional thoughts), mutism, anxiety, psychomotor agitation	Ectopia lentis, thrombopulmonary embolism	Acute	17 years
Kevere et al., 2014 [36]	116	15.56 years	MTHFR deficiency, hyperomocisteinemia	Schizophrenia-spectrum and mood disorders	Cognitive disturbances	Acute and chronic	/
Kumar, 2012 [37]	1	15 years	AIP	Psychotic symptoms (bizarre and disorganized thought and behavior)	None	Acute	14 years
Lahutte et al., 2008 [38]	3	15.7 years (range 14–17 years)	Storage disease	Catatonia, psychotic symptoms	/	Acute	/
Libernini et al., 2012 [39]	1	15 years	MNGIE	Mood disorder, anorexia nervosa symptoms	Hypotonia, diverticulosis of duodenum and ileum, muscular hypotrophy, reduced deep tendon reflexes, claw feet, hyporexia, weight loss, emesis	Chronic	15 years
Mandoki and Sumner, 1994 [40]	1	9 years	HC	Aggressive behavior, personality disorder (explosive type), hallucinations	Stomachaches, headaches, sleep disturbances	Acute	9 years
Manti et al., 2020 [41]	28	19.9 years (range 5–44 years, SD 10.9 years)	PTPsd	Generalized anxiety disorder; depressive disorder; obsessive compulsive disorder, attention deficit hyperactivity disorder	Movement disorders, intellectual disability, autonomic dysfunction, sleep disorders, epilepsy, scoliosis	Chronic	/
Manti et al., 2016 [42]	46	22.6 years (range 12–44 years, SD 7.35)	Early-treated PKU	Generalized anxiety disorder, depressive disorder, personality disorder, specific phobia, selective mutism	None	Chronic	14.5 years (range 6–32 years)
Niwinski et al., 2021 [43]	1	15 years	OCT deficiency	Social anxiety disorder, pervasive developmental disorder, selective mutism, atypical anorexia nervosa	Protein aversion	Chronic	13 years
Pan et al., 2013 [44]	2	19 years-youth	GTPCH deficiency, MTHFR deficiency	Major depressive disorder, suicidal ideation, non-suicidal self-injury	/	Acute and Chronic	14 years
Rahmandar et al., 2014 [45]	1	13 years	CblC deficiency	Psychotic symptoms (hallucinations, altered mental status), anorexia symptoms	Seizures, ataxia, urinary infections	Acute	13 years
Roze et al., 2003 [46]	2	20 years (range 16–24 years)	CblC deficiency	Psychotic symptoms (delusions, dissociative symptoms, auditory and visual hallucinations)	Progressive neuropathy, urinary incontinence, unsteady gait, areflexic paraparesis	Chronic	16 years
Sandu et al., 2009 [47]	1	17 years	NPC	Psychotic symptoms (auditory and visual hallucinations, paranoid delusions), obsessive compulsive symptoms, aggressive behavior	Neonatal jaundice, developmental delay, absence epilepsy, dysdiadochokinesia, ataxia, dysarthria	Acute	16 years
Santosh et al., 1994 [48]	1	14 years	AIP	Psychosis, catatonia	Headache, intellectual disability, muteness, cramp-like epigastric pain, insomnia, echolalia	Acute	14 years
Serrano et al., 2009 [49]	9	10.6 years (range 4–27 years)	UCDs	Eating disorders, PICA, anxiety, confusion, disorientation, delirium-like state, behavioral and personality disorders, tic disorder	Gastrointestinal symptoms, protein aversion, cerebellar syndrome, loss of consciousness, coma, development delay, strabismus, hepatomegaly, autism traits	Acute and chronic	5,8 years (range 2–15 years)
Simons et al., 2006 [1]	53	Range 0–18 years	PKU, MDs, MMA, storage diseases, HGA, Homocystinuria, OCT deficiency	Behavior, mood and anxiety disorders, attention deficit hyperactivity disorder, oppositional defiant disorder	Cognitive impairment	Chronic	/
Smith et al., 1988 [50]	544	8 years	Early-treated PKU	Defiant behavior, hyperactivity, anxiety	/	Chronic	/
Srinivas et al., 2008 [51]	15	19.8 years (SD 5.8 years)	WD	Mood (both depressive and manic manifestations) and anxiety disorders, schizophreniform-like symptoms	Neuropathy, Parkinson’s disease, and cognitive decline	Acute	/
Tantawy et al., 2019 [52]	22	14.6 years (range 6–29 years, SD 6.2 years)	GD	Depression, generalized anxiety disorder, attention deficit hyperactivity disorder, schizophrenia-like disorder, suicidality, tic disorder	/	Chronic	2 years (range 1–10 years)
Valayannopoulos et al., 2012 [53]	6	7.1 years (range 2–16 years)	CTP deficiency due to SLC6A8 gene mutation	Psychotic symptoms (hallucinations), behavior disturbance, anxiety, hyperactivity	Myoclonic and absence epilepsy, intellectual disability, muscular hypotonia, autistic traits	Chronic	/
Weglage et al., 2000 [54]	42	14.7 years (range 10–18 years, SD 2.9)	PKU	Behavioral, mood and anxiety disorders	/	Chronic	/
Wouters et al., 2014 [55]	1	16 years	NPC	Psychotic symptoms (auditory and visual hallucinations, paranoid delusions), agitation, anxiety, hyperactivity	Cognitive regression, ataxia, swallowing problems, vertical supranuclear gaze palsy, splenomegaly	Acute	16 years

**Table 2:** Time of onset of psychiatric manifestations in patients affected by inherited metabolic diseases. MNGIE = mitochondrial neurogastrointestinal encephalomyopathy; PKU = phenylketonuria; and SIBs = self-injurious behaviors.

TIME OF ONSET	metabolic condition	psychiatric signs
Infancy–Early childhood: 1 month–5 years-old	PKU and biopterin metabolism errors Urea cycle disorders Methylmalonic and propionic acidemia Congenital disorders of glycosylation Lesch–Nyhan syndrome Gaucher disease	Aggressive behavior, oppositional defiant disorder, obsessive compulsive disorder, attention deficit hyperactivity disorder, mood and anxiety disorders, eating disorders, SIBs, tic disorder
Childhood–Adolescence: 5–15 years-old	PKU and biopterin metabolism errors Urea cycle disorders Porphyrias Mitochondrial disorders Cerebrotendinous xanthomatosis Homocystinuria Creatine transporter deficiency	Psychosis, mood and anxiety disorders, catatonia, eating disorders, aggressive behavior, oppositional defiant disorder, obsessive compulsive disorder, attention deficit hyperactivity disorder
Late Adolescence: 15–18 years-old	PKU and biopterin metabolism errors Cerebrotendinous xanthomatosis Homocystinuria Niemann–Pick type C Wilson disease MNGIE	Psychosis, mood and anxiety disorders, catatonia, eating disorders, aggressive behavior

**Table 3:** Psychiatric phenomenology of inborn errors of the metabolism in children and adolescents. BH4 = tetrahydrobiopterin; cblC = cobalamin C disorder; CBSd = cystathionine β-synthase deficiency; CDG = congenital disorders of glycosylation; CTX = cerebrotendinous xanthomatosis; GD = Gaucher disease; HGA = hydroxyglutaric aciduria; LNS = Lesch–Nyhan syndrome; MDs = mitochondrial disorders; MMA = methylmalonic acidemia; MNGIE = mitochondrial neurogastrointestinal encephalomyopathy; MTHFR = methylenetetrahydrofolate reductase; NPC = Niemann–Pick type C disease; PA = propionic acidemia; PKU = phenylketonuria; PTPsd = 6-pyruvoyl-tetrahydropterin synthase deficiency; UCDs = urea cycle defects; and WD = Wilson’s disease.

	MOOD AND ANXIETY DISORDERS	SCHIZOPHRENIA-SPECTRUM DISORDERS	CATATONIA	EATING DISORDERS	SELF-INJURIOUS BEHAVIORS
Inborn errors of the metabolism (IEMs)	PKU BH4 deficiencies L-2 HGA MTHFR deficiency CBSd MDs CTX GD WD CDG Acute porphirias NPC	Acute porphirias CTX NPC CBSd MTHFR deficiency CblC WD UCDs MDs	Acute porphirias CTX Lysosomal storage diseases WD UCDs Homocysteine metabolism disorders	UCDs MMA PA MNGIE	LNS PKU PTPsd Hepatolenticular degeneration

**Table 4:** Inherited metabolic diseases that may present with schizophrenia-spectrum disorders in children and adolescents.

DISEASES	PSYCHIATRIC SIGNS	NEUROLOGICAL SIGNS	SYSTEMIC SIGNS
ACUTE PORPHYRIAS	Psychosis, mood disorders (namely depressive type), confusion, catatonia.	Acute peripheral neuropathy, dysautonomia, epilepsy.	Severe abdominal pain, nausea, emesis, and constipation. Cutaneous signs.
CEREBROTENDINOUS XANTHOMATOSIS	Schizophreniform attacks. Attention deficit hyperactivity disorder.	Progressive spastic paraparesis, cerebellar ataxia, polyneuropathy. Epilepsy, cognitive impairment, dementia.	Xanthomas, juvenile cataract, chronic diarrhea.
NIEMANN–PICK TYPE C DISEASE	Psychosis, behavioral disorders, depression, autism-spectrum-like disorders.	Supranuclear gaze palsy. Progressive ataxia, epilepsy, spasticity, dysarthria, dysphagia, deafness, cataplexy.	Hepatosplenomegaly. Cherry-red spot on the macula or gray pigmentation around the fovea in the fundus oculi.
HOMOCYSTEINE METABOLISM DISORDERS	Personality and behavioral (aggressiveness) disorders, depression, obsessive compulsive disorder, schizophrenic-like disorders.	Focal neurological signs, epilepsy, movement disorders, peripheral neuropathy, pyramidal signs, strokes.	Thromboembolic events. Lenticular ectopia, severe myopia, optic atrophy. Atherothrombotic disease.
UREA CYCLE DISORDERS	Mood disorders or psychosis, eating disorders.	Stroke-like episodes (diplopia, hemiparesis) coma, epilepsy, pyramidal signs.	Nausea, vomiting, and headache.
WILSON’S DISEASE	Mood disorders (depressive and manic manifestations), personality changes, aggressive behavior, schizophrenia-like presentation, catatonia.	Dysarthria, ataxia, hypomimia and motor clumsiness. Spasticity, seizures, gait, and movement disorders. Dysphagia and autonomic dysfunction.	Corneal Kayser–Fleischer ring. Chronic liver disease.

**Table 5:** Diagnostic chart for clinicians. MNGIE = mitochondrial neurogastrointestinal encephalomyopathy; PKU = phenylketonuria; and SIBs = self-injurious behaviors.

	1 MONTH–5 YEAR-OLD		5–15-YEAR-OLD		15–18-YEAR-OLD	
	IEMs	Red Flags/Other Manifestations	IEMs	Red Flags/Other Manifestations	IEMs	Red Flags/Other Manifestations
MOOD AND ANXIETY DISORDERS	PKU and biopterin metabolism errors; congenital disorders of glycosylation; Gaucher disease	Developmental delay; blonde hair; blue eyes; fair complexion; hypotonia; protein-losing enteropathy; hepatic involvement; skeletal abnormalities	PKU and biopterin metabolism errors; porphyrias; mitochondrial disorders; cerebrotendinous xanthomatosis; homocystinuria; creatine transporter deficiency; hydroxyglutaric aciduria	Executive functions’ deficit; intellectual disability; learning disorders; cerebellar signs; movement disorders; autonomic dysfunction; abdominal pain; nausea; emesis; cutaneous signs; epilepsy; neuropathy; xanthomas; juvenile cataract; thromboembolic events; autism traits	PKU and biopterin metabolism errors; cerebrotendinous xanthomatosis; homocystinuria; Niemann–Pick type C; Wilson disease	Movement disorder; intellectual disability; autonomic dysfunction; epilepsy; executive functions deficit; supranuclear gaze palsy; hepatosplenomegaly; xanthomas; juvenile cataract; thromboembolic events; corneal Kayser–Fleischer ring; chronic liver disease
SCHIZOPHRENIA-SPECTRUM DISORDERS			Urea cycle disorders; porphyrias; mitochondrial disorders; cerebrotendinous xanthomatosis; homocystinuria; creatine transporter deficiency	Stroke-like episodes; epilepsy, pyramidal signs; nausea; emesis; autonomic dysfunction; abdominal pain; cutaneous signs; xanthomas; juvenile cataract; thromboembolic events; autism traits	Cerebrotendinous xanthomatosis; homocystinuria; Niemann–Pick type C; Wilson disease	Supranuclear gaze palsy; hepatosplenomegaly; xanthomas; juvenile cataract; thromboembolic events; corneal Kayser–Fleischer ring; chronic liver disease; cerebellar signs; movement disorders; autonomic dysfunction
CATATONIA			Urea cycle disorders; porphyrias; cerebrotendinous xanthomatosis; homocystinuria	Stroke-like episodes; epilepsy; pyramidal signs; nausea; emesis; autonomic dysfunction; abdominal pain; cutaneous signs; xanthomas; juvenile cataract; thromboembolic events	Cerebrotendinous xanthomatosis; homocystinuria; Wilson disease	Xanthomas; juvenile cataract; thromboembolic events; corneal Kayser–Fleischer ring; chronic liver disease; cerebellar signs; movement disorders; autonomic dysfunction
EATING DISORDERS	Urea cycle disorders; methylmalonic and propionic acidemia	Protein aversion; emesis; altered consciousness; developmental delay; hypotonia; gastrointestinal symptoms; cerebellar syndrome; strabismus; hepatomegaly; autism traits	Urea cycle disorders	Protein aversion; emesis; altered consciousness; hypotonia; gastrointestinal symptoms; cerebellar syndrome; strabismus; hepatomegaly; autism traits	MNGIE	Hypotonia; duodenum and ileum diverticulosis; muscular hypotrophy; reduced deep-tendon reflexes; claw feet; hyporexia; weight loss; emesis
SIBs	PKU and biopterin metabolism errors; Lesch–Nyhan syndrome	Developmental delay; teething and renal problems				

## Data Availability

The data presented in this study are available on request from the corresponding author (accurately indicate status).
